# Integrated HIV surveillance finds recent adult hepatitis B virus (HBV) transmission and intermediate HBV prevalence among military in uncharacterized Caribbean country

**DOI:** 10.1371/journal.pone.0222835

**Published:** 2019-10-01

**Authors:** Siobhan M. O’Connor, Tonya Mixson-Hayden, Lilia Ganova-Raeva, Djeneba Audrey Djibo, Matthew Brown, Guo-Liang Xia, Saleem Kamili, Marni Jacobs, Maxia Dong, Anne G. Thomas, Marc Bulterys, Braden Hale

**Affiliations:** 1 Division of Viral Hepatitis, National Center for HIV/AIDS, Viral Hepatitis, STD and TB Prevention, United States Centers for Disease Control and Prevention, Atlanta, GA, United States of America; 2 Department of Defense HIV/AIDS Prevention Program, United States Naval Health Research Center, San Diego, CA, United States of America; 3 Division of Global HIV and Tuberculosis, Center for Global Health, United States Centers for Disease Control and Prevention, Atlanta, GA, United States of America; 4 Division of Global HIV and Tuberculosis, United States Centers for Disease Control and Prevention, CDC Guyana, Georgetown, Guyana; University of Cincinnati College of Medicine, UNITED STATES

## Abstract

**Background:**

Guyana expanded its HIV response in 2005 but the epidemiology of hepatitis B virus (HBV) and hepatitis C virus (HCV) infections has not been characterized.

**Methods:**

The 2011 Seroprevalence and Behavioral Epidemiology Risk Survey for HIV and STIs collected biologic specimens with demographic and behavioral data from a representative sample of Guyana military personnel. Diagnostics included commercial serum: HIV antibody; total antibody to hepatitis B core (anti-HBc); IgM anti-HBc; hepatitis B surface antigen (HBsAg); anti-HBs; antibody to HCV with confirmatory testing; and HBV DNA sequencing with S gene fragment phylogenetic analysis. Chi-square, p-values and prevalence ratios determined statistical significance.

**Results:**

Among 480 participants providing serologic specimens, 176 (36.7%) tested anti-HBc-positive. Overall, 19 (4.0%) participants tested HBsAg-positive; 17 (89.5%) of the HBsAg-positive participants also had detectable anti-HBc, including 1 (5.3%) IgM anti-HBc-positive male. Four (6.8%) females with available HBV testing were HBsAg-positive, all aged 23–29 years. Sixteen (16, 84.2%) HBsAg-positive participants had sufficient specimen for DNA testing. All 16 had detectable HBV DNA, 4 with viral load >2x10^4^IU/ml. Sequencing found: 12 genotype (gt) A1 with 99.9% genetic identity between 1 IgM anti-HBc-positive and 1 anti-HBc-negative; 2 gtD1; and 2 with insufficient specimen. No statistically significant associations between risk factors and HBV infection were identified.

**Conclusions:**

Integrated HIV surveillance identified likely recent adult HBV transmission, current HBV infection among females of reproductive age, moderate HBV infection prevalence (all gtA1 and D1), no HCV infections and low HIV frequency among Guyana military personnel. Integrated HIV surveillance helped characterize HBV and HCV epidemiology, including probable recent transmission, prompting targeted responses to control ongoing HBV transmission and examination of hepatitis B vaccine policies.

## Introduction

The Cooperative Republic of Guyana (Guyana) in the Caribbean region (northeast South America), was heavily impacted by the human immunodeficiency virus (HIV) epidemic [1–2]. In 2003, an estimated 2.5% of the general population, including pregnant women, were infected with HIV [3]; and by 2006, HIV/AIDS was the leading cause of death among persons aged 15–44 years (who comprised >50% of the population) [4]. Following expansion of HIV prevention and treatment efforts, 2011 Guyana general population-based surveillance estimated a decrease in HIV prevalence to 1.1% among persons aged 15–49 years [4–6]. The 2008–2009 national behavioral survey included military personnel and identified gaps in HIV prevention and knowledge among military but did not include biologic testing for infections [4]. In general, military populations are at increased risk for sexually transmitted infections (STIs) [7–9].

The epidemiology of hepatitis B virus (HBV) and hepatitis C virus (HCV) infections in Guyana is not well characterized [10–11]. However, an estimated 2–4% of persons in Latin America and the Caribbean (LAC) are infected with HBV. Rates range from <2% to >8%, even 10–30% among indigenous peoples, and genotypes (gt) associated with severe disease are reported [10–17]. Furthermore, an estimated 7–9 million persons in LAC live with chronic HCV infection. Moderate HCV infection prevalence is reported in countries bordering Guyana but published Caribbean-specific HCV infection epidemiology and gt data are limited [10–11, 18–19].

Understanding the epidemiology of HBV and HCV infections in Guyana is important. Globally, HBV- and HCV-related deaths have been increasing over time. HBV- and HCV- related chronic liver disease and hepatocellular cancer accounted for an estimated 720,000 and 470,000 deaths in 2015 [10, 20]. Furthermore, HBV and HCV are opportunistic co-infections with HIV that may negatively impact HIV outcomes [21–25] so a good understanding of HBV and HCV treatment needs within HIV programs is essential. This report describes the first effort to define the epidemiology of HBV and HCV infections and co-infections with HIV in the Guyana general population or military forces, with associated risk factors, by integrating HBV and HCV testing into HIV surveillance among military personnel. The data can inform prevention and control policy and programs.

## Methods

### Study design and population

The Seroprevalence and Behavioral Epidemiology Risk Survey (SABERS) implements cross-sectional behavioral risk questionnaires paired with biologic specimen collection for HIV and STI (not previously including viral hepatitis) testing from representative members of military forces in lower resourced nations [26]. The 2011 Guyana Defence Force (GDF) SABERS incorporated testing for acute and chronic HBV infection plus evidence of HCV exposure and infection. Briefly, the GDF invited all active military personnel aged ≥18 years (yr) to participate. Using a stratified proportional sampling scheme of all military personnel based on rank distribution at each base, the survey target sample size of 500 was calculated to represent 20% of the total active military population and fulfill the primary SABERS objective, to determine HIV prevalence in the combined military forces. Sampling assumed a general population HIV prevalence rate of 2.4% (95% confidence interval [CI] 0.8%– 4.1%) [27]. Female personnel were separately recruited to compose 10% of the sample, equivalent to the proportion of females in the Forces. Of note, females are not stationed in remote areas.

This study was approved by the institutional review boards of the Guyana Ministry of Health and Naval Health Research Center (NHRC) in San Diego, California.

### Collection of demographics, behavioral history and biologic specimens

All participants provided their written free and informed consent to participate and give biologic specimens. Trained survey staff provided pre-survey counseling and proctored participants, who self-administered the written behavioral risk survey. To protect anonymity, each participant was assigned a randomly generated numerical code not linked to names. The questionnaire adapted existing SABERS and Guyana Demographic and Health Survey forms to collect demographics, military service history and self-reported HIV risk behaviors [26–27]. Questions about risk behaviors during the preceding 12 months included queries about alcohol use, illicit drug use and injection drug use (IDU), sexual preference, and sexual practices and experiences. Trained phlebotomists collected venous blood for HIV, HBV, HCV and syphilis testing and participants provided urine for chlamydia and gonorrhea analysis. The Force Medical Officer provided test results and post-test counseling to participants with the corresponding numerical code and referred infected participants to standard care in the civilian sector. This report outlines only the HBV, HCV and HIV test results and risk factors associated with those infections.

### Laboratory testing

HIV testing followed the existing Guyana national testing guidelines [28]. The Guyana National Public Health Reference Laboratory performed parallel, dual rapid testing for antibody to HIV (Inverness Medical Determine HIV-1/2 Ag/Ab Combo [Alere], Inverness Medical Innovations, Inc., Waltham, MA; and Trinity Biotech Unigold Recombigen, Trinity Biotech PLC, Bray, Ireland) with a tie-breaker test (Chembio Stat-Pak, ChemBio Diagnostic Systems, Inc., Medford, NY) for discordant results. Venous blood was processed and serum frozen. Stored serum was shipped to the CDC Division of Viral Hepatitis for hepatitis B surface antigen (HBsAg), total antibody to hepatitis B core antigen (total anti-HBc), antibody to hepatitis B surface antigen (anti-HBs), immunoglobulin M (IgM) anti-HBc as indicated, and antibody to HCV (anti-HCV) testing on an automated platform (Ortho VITROS ECi, Ortho Clinical Diagnostics, Inc., Raritan, NJ). All available participant sera underwent testing for total anti-HBc, HBsAg and anti-HBs. Those with detectable HBsAg (HBsAg-positive) were further tested for IgM anti-HBc and underwent polymerase chain reaction testing (COBAS^®^ AmpliPrep^®^/COBAS^®^ TaqMan^®^ HBV v2.0, Roche Diagnostics, Indianapolis, IN) with quantification of HBV viral load (HBV VL). Specimens reactive for anti-HCV (anti-HCV-positive) underwent still available recombinant immunoblot assay (RIBA, CHIRON^®^ RIBA^®^ HCV 3.0 SIA, Novartis Vaccines and Diagnostics, Inc., Emeryville, CA); and RIBA-positive specimens were further tested for HCV RNA (COBAS^®^ TaqMan^®^ HCV Test v2, Roche Diagnostics, Indianapolis, IN).

### Phylogenetic analysis

Specimens with detectable HBV DNA underwent full genome sequencing with phylogenetic analysis of an S gene fragment and characterization of genetic identity [29–31]. Nucleotide sequences were aligned using the Geneious (Version 7.1.5, Biomatters Limited, Auckland, New Zealand) Align/Assemble program. HBV gt was classified based on the S-gene sequence and confirmed with whole-genome sequences by comparing each sequence with published reference sequences from GenBank. Initial neighbor-joining trees were built using the Kimura two-parameter model of nucleotide substitution [31]. Phylogenetic trees were constructed using the maximum likelihood algorithm implemented in DNAML (PHYLIP package, v.3.6).

### Classification of infection

Two concordant positive HIV screening tests or a positive HIV tie-breaker test signified HIV infection (HIV-positive). HBsAg-positive results indicated active HBV infection. Detection of IgM anti-HBc (IgM anti-HBc-positive) signified acute, recent HBV infection within the 6 months preceding venipuncture. Detection of anti-HBs (anti-HBs-positive) in the absence of HBsAg defined either HBV immune status after resolved infection (anti-HBs-positive/total anti-HBc-positive) or previous hepatitis B immunization (anti-HBs-positive/total anti-HBc-negative). Detection of isolated total anti-HBc in the absence of other detectable HBV markers was classified as indeterminate infection. Confirmation of anti-HCV-positive status by RIBA indicated HCV infection at some time but only detection of HCV RNA (HCV RNA-positive results) signified current HCV infection.

### Statistical analysis

Trained staff double entered information into Epi Info^™^ 7 (CDC, Atlanta, GA). Denominators for HBV and HCV infection frequencies equaled the number of participants having sufficient specimen to perform both HBsAg and anti-HBc testing, or anti-HCV testing, respectively, and frequency comparisons were restricted to those participants; behavioral analyses reflected participants having both HBV results and a completed questionnaire for that risk factor. Statistical analyses were performed using SAS Version 9.3 (SAS Institute, Cary, NC) and Epi Info^™^ 7. Chi-square test for independence, p-values generated using the Fisher exact test or two sample z-test comparison of proportions and prevalence ratios (PR) with 95% confidence intervals (CI) derived using maximum likelihood estimates determined statistical significance.

## Results

### HBV and HCV testing

**Fig 1:**
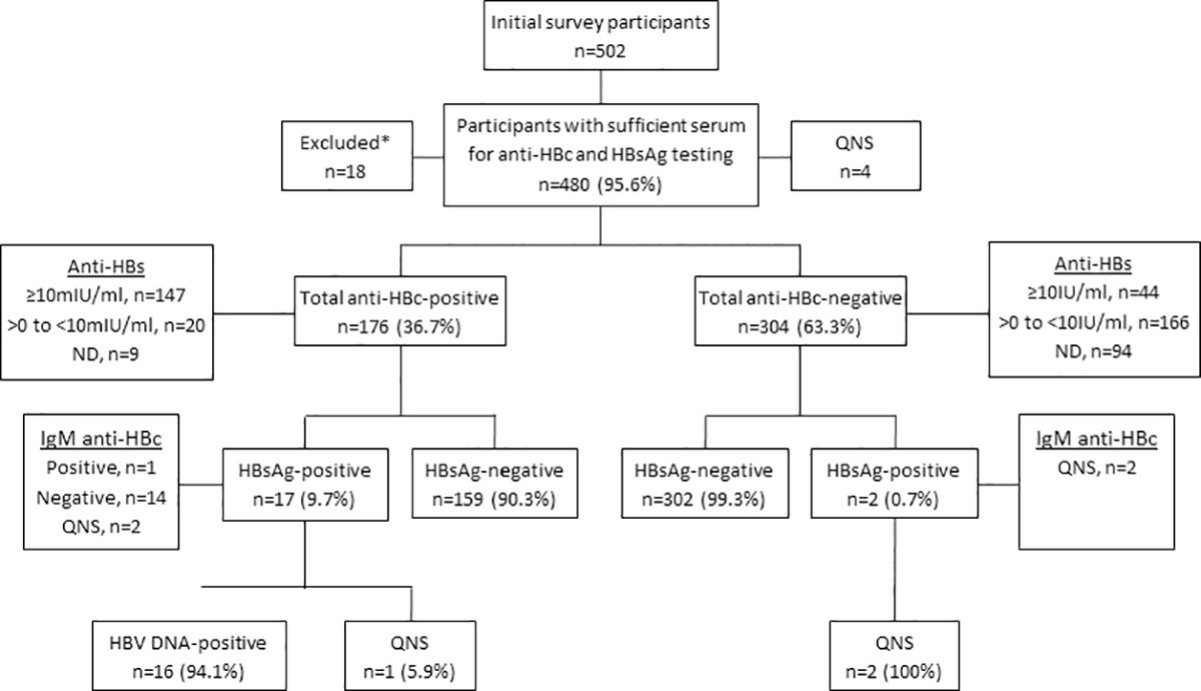
Hepatitis B virus (HBV) testing with results, 2011 Guyana Defense Force Seroprevalence and Behavioral Epidemiology Risk Survey for HIV and sexually transmitted infections. Abbreviations: Anti-HBc, antibody to hepatitis B core antigen; Anti-HBs, antibody to hepatitis B surface antigen; DNA, deoxyribonucleic acid; HBsAg, hepatitis B surface antigen; HBV, hepatitis B virus; IgM, immunoglobulin M; mIU/ml, milli-international units per milliliter; ND, not detected; QNS, quantity not sufficient for testing.

Among 502 total participants, 480 (95.6%) had sufficient serum for both HBsAg and total anti-HBc testing; the HBV frequency denominator is defined as the number of participants with results for both HBsAg and anti-HBc testing. Of the 480, 176 (36.7%) tested total anti-HBc-positive. While 19 (4.0%) tested HBsAg-positive, only 17 (89.5%) HBsAg-positives had detectable anti-HBc, including 1 (5.3%) IgM anti-HBc-positive male (Fig 1). Frequency of HBsAg detection by sex was not statistically different (males 3.7% vs. females 6.8%, p = 0.20). Of the 19 HBsAg-positive participants, 17 (89.5%) were aged 20-34yr (Table 1), statistically similar to the survey age distribution (S1 Table). All 4 HBsAg-positive females were aged 20-29yr; although younger than male counterparts, the difference was not statistically significant. Among the 19 HBsAg-positive participants, 15 had sufficient specimen for IgM anti-HBc testing, with only 1 male having detectable IgM anti-HBc (3 males and 1 female had insufficient specimen). Overall, 103 (21.4%) of participants with HBV test results had undetectable anti-HBs. This included 95 (20.6%) HBsAg-negative/anti-HBc-negative participants, while 177 (38.4%) of all HBsAg-negative participants demonstrated anti-HBs levels <10 mIU/mL.

**Table 1:** Serology and genetic identity of hepatitis B virus infections identified among military forces in the 2011 Guyana Defence Force Seroprevalence and Behavioral Epidemiology Risk Survey for HIV and sexually transmitted infections (n = 19).

Genetic identity, S gene		Sex	Age (years)	Total anti-HBc	IgM anti-HBc	HBsAg	HBV DNA(IU/mL)	Anti-HBs (mIU/mL)	Anti-HCV	HBV Genotype
99.1 to 100%		M	31	Pos	Pos	Pos	305	0.6	Neg	A1 ^CG^
		M	29	Pos	Neg	Pos	334	3.6	Neg	A1 ^CG^
		M	34	Pos	Neg	Pos	6,196	0	Neg	D
		M	37	Pos	Neg	Pos	5,321	1.1	Neg	D
98.1 to <99.1%		F	23	Pos	Neg	Pos	20,670	0.8	Neg	A1 ^CG^
		F	29	Pos	Neg	Pos	500	1.0	Neg	A1 ^CG^
		M	34	Pos	Neg	Pos	2,804	1.0	Neg	A1 ^CG^
		M	25	Pos	Neg	Pos	138	0	Neg	A1 ^CG^
<98.1%		F	23	Pos	Neg	Pos	69,774,024	0	Neg	A1
		F	24	Neg	QNS	Pos	QNS	2.6	Neg	QNS
		M	19	Pos	Neg	Pos	133,259670	86.2	Neg	A1
		M	31	Pos	QNS	Pos	116,940	0	Neg	A1-3/5
		M	24	Pos	Neg	Pos	18,330	0	Neg	A1
		M	26	Pos	QNS	Pos	6,053	0	Neg	*
		M	21	Pos	Neg	Pos	485	13.5	Neg	A1
		M	26	Pos	Neg	Pos	31	1.5	Neg	*
		M	34	Pos	Neg	Pos	195	0	Neg	A1
		M	34	Neg	QNS	Pos	QNS	0	Neg	QNS
		M	22	Pos	Neg	Pos	QNS	0.5	Neg	QNS

*Unable to amplify

Abbreviations: Anti-HBc, antibody to hepatitis B core antigen; anti-HBs, antibody to hepatitis B surface antigen; anti-HCV, antibody to hepatitis C virus; ^CG^complete genome sequence available; HBsAg, hepatitis B surface antigen; HBV, hepatitis B virus; HBV DNA, hepatitis B virus deoxyribonucleic acid; IgM anti-HBc, immunoglobulin M anti-HBc; IU/ml, international units per milliliter; mIU/ml, milli-IU/ml; N/A, not applicable; Neg, negative; Pos, positive; QNS, quantity not sufficient.

Sixteen (16) HBsAg-positive participants had sufficient specimen for DNA testing; all 16 were HBV DNA-positive. VLs varied between 3.10 x 10^1^ IU/ml and 1.33 x 10^9^ IU/ml; and 4 (25.0%) had HBV VL >2.00 x 10^4^ IU/ml, including 1 female with VL 6.98 x 10^8^. Of the 16, 2 (12.5%) had anti-HBs titers ≥10mIU/ml (12.5 and 86.2mIU/ML), with HBV VL 1.33 x 10^9^ and 4.85 x 10^2^, respectively.

Testing detected no current HCV infections. Four (0.8%) individuals initially tested anti-HCV-positive but HCV infection could not be confirmed: 2 RIBA-negative; and 2 RIBA-indeterminate with no detectable HCV RNA.

### HBV phylogenetic analysis

**Fig 2:**
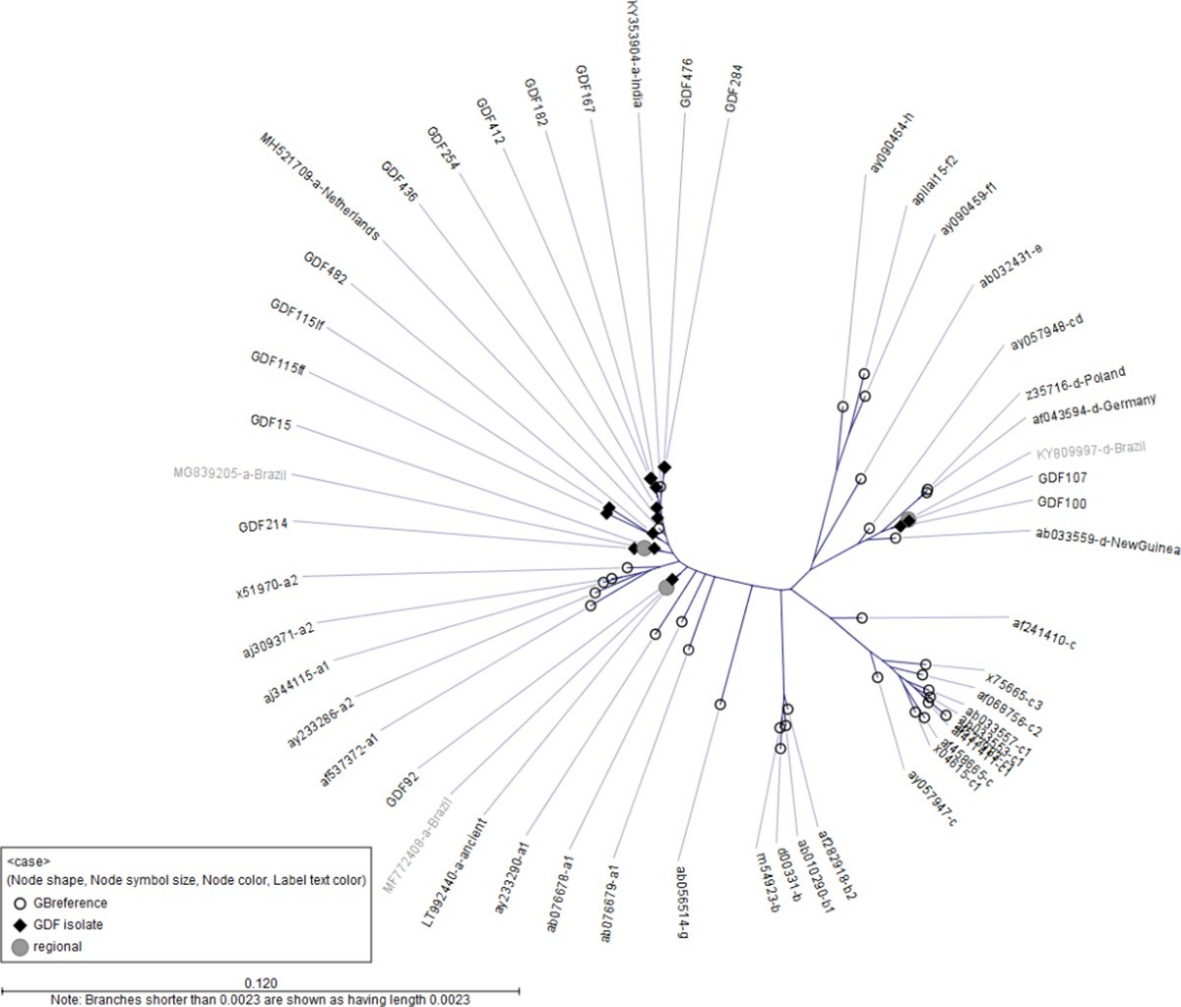
Genetic relatedness of hepatitis B virus cases as demonstrated with phylogenetic maximum likelihood tree constructed using S gene sequences, 2011 Guyana Defense Force Seroprevalence and Behavioral Epidemiology Risk Survey for HIV and sexually transmitted infections.

Genotyping by S gene sequencing of 14 HBV DNA-positive participants with sufficient specimen identified 12 (85.7%) gtA1 cases, in which 1 case had 2 gtA1 variants, and 2 (14.3%) gtD1 cases. The 2 gtD1 infections were genetically identical in the S gene region (Fig 2), as were 4 gtA1 cases. Complete genome sequencing was successful for 6 of the genotyped cases (Table 1). Two males and 1 female with gtA1 infections demonstrated ≥98.6% sequence identity at the complete genome level as well as the S gene.

0.005 = sequence distance unit (number of changes per 100 nucleotides); sequence names starting with GDF are HBV DNA-positive cases and all other notations represent reference sequences with corresponding GenBank number and genotype.

### HIV testing

Only 1 participant tested HIV-positive, without evidence of HBV or HCV co-infection.

### Population characteristics, behavioral survey results and correlation with laboratory results

Combined, 469 participants (410 males and 59 females) with HBV testing submitted questionnaires, including all HBsAg-positive participants. The denominators to calculate risk factor frequency equaled the number of participants who responded to each specific risk factor question. The age distribution of participants completing questionnaires was as follows: 9.7% (18-19yr); 45.3% (20-24yr); 23.4% (25-29yr); 14.9% (30-34yr); 4.7% (35-39yr); and 3.0% (>40yr). Seventeen (89.5%) HBsAg-positive participants self-identified as Afro-Guyanese and 2 (10.5%) as mixed race, statistically similar to the survey population composition (prevalence ratio [PR] 1.4, 95% CI, 0.5–4.2) (Table 2). Most (70.8%) were born in populated coastal areas and only 3.5% in the non-coastal and remote hinterland (densely forested, jungle) regions; HBsAg-positive frequency did not differ by region of birth (PR 3.2, 95% CI, 0.4–24.0). Only 1 HBsAg-positive participant reported IDU compared with 5 HBsAg-negative participants (PR 4.4, 95% CI, 0.7–27.8).

**Table 2:** Frequency of self-reported demographic and behavioral risk factors by hepatitis B virus infection status among military forces, 2011 Guyana Defence Force Seroprevalence and Behavioral Epidemiology Risk Survey for HIV and sexually transmitted infections.

	Males and females combined			
		Risk factor frequency by HBsAg status n (% of HBsAg-positive or -negative)		
Risk factor	Number of responses^a^ n	HBsAg-positive	HBsAg-negative	Prevalence ratio (95% CI)
Race/ethnicity	439			
Afro-Guyanese		15 (78.9)	304 (72.4)	1.4 (0.5–4.2)
Other		4 (21.1)	116 (27.6)	1.0
Birth region	451			
Coastal^b^		18 (94.7)	364 (84.3)	3.2 (0.4–24.0)
Non-coastal and remote hinterlands^c^		1 (5.3)	68 (15.7)	1.0
Ever stationed remotely	466	7 (36.8)	189 (42.3)	0.9 (0.5–1.5)
Illicit drug us	418	1 (6.3)	40 (10.0)	0.6 (0.1–4.5)
Injection drug use	466	1 (5.3)	5(1.1)	4.4 (0.7–27.8)
Lifetime number of sexual partners	425	6.8 (4.1)	8.2 (9.2)	
≤4		8 (47.1)	115 (28.2)	1.3 (0.4–4.8)
5–10		6 (35.3)	235 (57.6)	0.5 (0.1–2.0)
≥11+		3 (17.6)	58 (14.2)	1.0
Inconsistent condom use with regular sexual partner^d^	402	15 (79.0)	289 (75.5)	1.2 (0.4–3.6)
Inconsistent condom use with non-regular sexual partner^e^	158	5 (62.5)	96 (64.0)	0.9 (0.2–3.8)

^a^Analysis restricted to number of participants who responded to each question.

^b^Coastal regions by number and name: 2 (Pomerron-Supenaam), 3 (Essequibo Islands–West Demerara), 4 (Demerara-Mahaica), 5 (Mahaica-Berbice), 6 (East Berbice–Corentyne).

^c^Non-coastal and remote hinterland regions by number and name: 1 (Barima-Waini), 7 (Cuyuni-Mazaruni), 8 (Potaro-Siparuni), 9 (Upper Takatu-Upper Essequibo), 10 (Upper Demerara-Berbice).

^d^Analysis further restricted to participants reporting regular sexual partners during the preceding 12 months.

^e^Analysis further restricted to participants reporting non-regular sexual partners during the preceding 12 months.

Abbreviations: CI, confidence interval; HBsAg, hepatitis B surface antigen

Among 158 participants reporting non-regular sexual partners during the preceding 12 months, the prevalence ratio did not suggest an association between unprotected sex and HBV infection (Table 2). However, only 39% reported knowing the HIV status of their non-regular partner. Similarly, of 87 participants reporting one or more transactional partners, only 59% reported using a condom at last sex and 48% at every encounter with those partners; just 41% of this group reported knowing the HIV status of the transactional partner. Across all respondents, HBV infection was not associated with number of sexual partners or unprotected sex (Table 2). Only 1 participant reported same sex partners. Twenty-two percent of males had been circumcised (at median age 10yr) and another 36% reported willingness to get circumcised. Notably, 7.9% of respondents believed that HIV could be transmitted by sharing a meal with an infected person and 12.0% were not sure if this was possible; 4.3% thought HIV could be transmitted by mosquito bites, while another 17.5% did not know if mosquito transmission was possible.

## Discussion

Integrating HBV and HCV testing into the 2011 GDF SABERS HIV surveillance provided the first indication of intermediate HBV and low HCV infection prevalence in any segment of the Guyana population. It also identified recent, acute adult HBV infection with likely HBV transmission among military personnel; the >99.1% genetic identity between 2 participant infections, including 1 IgM anti-HBc-positive male, is consistent with both a common source of infection and recent infection. We cannot, however, differentiate between direct person-to-person transmission and transmission through a common intermediary such as a shared infected sexual partner, shared personal hygiene implements or a close household contact. Living conditions in the remote military camps were rudimentary and lapses in hygiene, such as sharing of razors, toothbrushes or other personal implements, cannot be ruled out; suspected adult HBV and HCV transmission through shared razors at community barbers has been reported elsewhere [32, 33]. Although IDU behaviors might be under-reported, Guyana health officials indicate that IDU is uncommon in Guyana. No recent local viral hepatitis transmission through transfusions, lapses in infection control, unsafe injections or cultural practices (breaching the skin) that might explain the findings have been recognized.

The >98.1% genetic identity among >30% of the remaining gtA1 HBV infections suggests closely related sources. Those infections were not recent but this survey cannot determine whether HBV infection occurred during early childhood or later yet >6 months before the survey. The anonymity of SABERS methodology prevents determining whether participants were related by parentage or co-habitation (potential perinatal or household transmission). Nevertheless, the findings indicate that recent transmission with acute infection(s) occurred and could continue.

Two other findings have important implications for hepatitis B prevention and care programs and policy. Fully 6.8% of participating females–all of reproductive age–evidenced chronic HBV infection, 1 with VL>6.9x10^7^ IU/ml, representing high risk for mother-to-child HBV transmission if pregnancy occurred; but the Guyana immunization schedule does not include routine (universal) hepatitis B vaccine birth dose or hepatitis B immune globulin for optimal prevention of mother-to-child HBV transmission [34–35]. Additionally, 20.6% of participants demonstrated no immunity to HBV infection and another 38.4% were possibly not protected (low anti-HBs titers). They represent a substantial proportion of the military and an age cohort not captured by the high-performing Guyana routine childhood immunization program, which has included the routine hepatitis B vaccine series since 2001 [36]. Therefore this cohort represents a reservoir of susceptibles at risk for future HBV infection and ongoing transmission. Additionally, World Health Organization guidelines recommend that all HBsAg-positive individuals should be assessed for cirrhosis and counseled regarding risk for transmission to others [37].

Detection of only 1 HIV-infected individual suggests that HIV education and prevention efforts have had an impact. However, participant questionnaire responses indicate substantial lapses in safe sexual behaviors and misconceptions about HIV transmission among the military personnel. Paired with rising STI rates among Guyanese adults and youths when the survey was conducted [3, 4], the findings indicate that further HIV and STI education was still needed. Progress in the Guyana response to its HIV/AIDS epidemic was reported elsewhere by the Republic of Guyana [38].

The survey findings are subject to several limitations. The results and conclusions of a survey among military personnel might not be generalized to the general population. Furthermore, the small sample size and voluntary nature of participation could have introduced bias into sampling, underestimating HIV, HBV and HCV infection prevalence. Individuals already aware of their HIV status, particularly those who are HIV-infected, may have chosen not to participate because they did not perceive personal value in participating. Although the GDF has a liberal policy of HIV tolerance, and HIV infection status does not impact service eligibility or advancement, fear of public knowledge of personal HIV status might still have deterred the participation of HIV-infected staff. Lastly, underreporting of IDU and sexual risk behaviors might have occurred, and the survey did not include mechanisms to verify the responses.

In conclusion, the addition of HBV and HCV testing to HIV surveillance in the 2011 GDF SABERS provided actionable public health findings with implications for viral hepatitis prevention and care. The addition of IgM and total anti-HBc, anti-HBs, HBV DNA and HBV phylogenetic analysis to HBsAg testing identified likely ongoing recent HBV transmission between adult military personnel, active HBV infection among women of reproductive age and a high frequency of HBV susceptibility in the survey population. Although not necessarily representative of the general population of Guyana, the findings still provide important insight into HBV and HCV infection prevalence, risk and, importantly, the potential for preventable mother-to-child HBV transmission in this previously uncharacterized region of LAC. In particular, high HBV infection rates and HBV VL in females of reproductive age highlight the need to address national immunization policies and antenatal care practice, particularly the addition of universal hepatitis B vaccine birth dose delivery, HBsAg testing of pregnant females and hepatitis B vaccination of susceptible young adults. It also highlights the need for hepatitis B vaccination among military personnel, particularly in the setting of known recent transmission, and inclusion of females of reproductive age [34–35]. In response to the findings, the GDF began implementing measures to interrupt and prevent further HBV transmission.

## Supporting information

S1 Table Age distribution by hepatitis B virus infection status among military forces, 2011 Guyana Defence Force Seroprevalence and Behavioral Epidemiology Risk Survey for HIV and sexually transmitted infections. (DOCX)
