# Supplementary material for: Integrated HIV surveillance finds recent adult hepatitis B virus (HBV) transmission and intermediate HBV prevalence among military in uncharacterized Caribbean country
Source: PLoS One. 2019 Oct 1;14(10):e0222835. doi: 10.1371/journal.pone.0222835 (PMC6772055; doi:10.1371/journal.pone.0222835)
Supplement: S1 Table — (DOCX) [file pone.0222835.s001.docx]

**S1 Table. Age distribution by hepatitis B virus infection status among military forces, 2011 Guyana Defence Force Seroprevalence and Behavioral Epidemiology Risk Survey for HIV and sexually transmitted infections**

| Age group (years) | N | Mean age (SD) |
| --- | --- | --- |
| HBsAg positive | 19 | 27.7 (5.3) |
| All survey participants | 502 | 25.6 (6.0) |

SD: Standard Deviation. Paired t-test p value >0.05.
